# Switch to pollution control bonds, else carbon risk will switch us: Evidence from the U.S. electric utility firms

**DOI:** 10.1016/j.heliyon.2024.e32487

**Published:** 2024-06-07

**Authors:** Imen Khanchel, Naima Lassoued, Cyrine Khiari

**Affiliations:** a Higher School of Business, Manouba University, Manouba, Tunisia; b LARIME LR11ES02, ESSECT, University of Tunis, Tunis, Tunisia; c QUARG UR17ES26, ESCT, Manouba University, Manouba, Tunisia

## Abstract

This study explores the implications and effectiveness of pollution control bonds (PCBs) in reducing carbon risk, focusing on a sample of 242 U S. electric utility firms from 2012 to 2022. The research investigates the association of PCB issuances with (i) absolute (unscaled) carbon emissions levels and their three scopes; and (ii) carbon emissions intensity and its three scopes. Using quantile regressions covering the 5th, 25th, 50th, 75th, and 90th quantiles, along with the Propensity Score Matching (PSM) methodology (where the treatment group includes firms using PCBs and the control group comprises firms that do not opt for PCBs), the findings show a significant reduction in carbon emissions due to PCB issuance. Substantial differences were observed between the treatment group and the control group across various quantiles of carbon emissions, particularly for companies with medium to high carbon footprints, both in terms of overall CO2 emissions and scope 2 CO2 emissions. Moreover, disparities between the two groups were notable across all quantiles of scope 1 CO2 emissions. Additionally, among the companies using PCBs, those with lower risk profiles exhibited a smaller carbon footprint, measured by scope 3 CO2 emissions, in comparison to their counterparts. Furthermore, the study highlights a more pronounced impact of PCBs issuance during the second phase of the Kyoto Protocol and the commitment period of the Paris Agreement. The results remain robust when alternative measures of carbon risk are applied. These findings carry significant implications for municipal and financing authorities, as well as investors within the U.S. electric utility industry. This research contributes novel insights to the field of electric utility management.

## 1 Introduction

Carbon risk, the potential financial impact of tightening carbon emissions policies, has garnered substantial attention from practitioners, policymakers, investors, and researchers. This issue is commonly described in literature as carbon dioxide (CO2) emissions [[1], [2], [3]]. Projections indicate that there will be a 7.6 % increase in global energy generation-induced CO2 emissions, reaching 43.2 billion metric tons by 2040 (International Energy Outlook, 2022), primarily driven by human activities [[4], [5], [6]]. The Greenhouse Gas Protocol (GHG) classifies CO2 emissions into three scopes: scope 1 (direct emissions), scope 2 (indirect emissions from purchased electricity generation), and scope 3 (indirect emissions from various value chains).

Over the past three decades, increasing CO2 emissions have directly contributed to climate change, necessitating a global commitment to addressing carbon risk and transitioning to a sustainable, low-carbon future. International agreements like the Kyoto Protocol (1997) and the Paris Agreement (2015) have underscored this commitment. Carbon pricing mechanisms, such as carbon trading and taxes, have been implemented in numerous countries, with 25 nations adopting carbon taxes (e.g., Canada, the U.K., Germany, Finland, and Singapore) and 36 embracing emissions trading schemes (e.g., the E.U., the U.S., Korea, and China). In this context, firms have adopted decarbonization strategies, including sustained carbon dependence [[7], [8], [9], [10]], carbon emissions compensation [8,11], carbon emissions mitigation [7,9,12], and carbon independence [8,13].

Despite extensive research on carbon risk determinants like governance [14], carbon disclosure quality [15], financial performance [[16], [17], [18]], cost of capital [[19], [20], [21]]), and eco-friendly investment [22], the impact of financing patterns, particularly green financing, on carbon risk remains not sufficiently explored [23].

This study addresses this gap by investigating how green financing can affect different forms of carbon risk using a sample of 242 U S. firms within the electric utility industry (121 firms utilizing PCBs and 121 matched non-PCB users) observed over the 2012–2022 period. Specifically, this study examines the impact of Pollution Control Bonds (PCBs) on carbon risk (overall CO2 emissions, scope 1 emissions, scope 2 emissions and scope 3 emissions) using the quantile estimation method.

The results show that electric utility companies using PCB financing experience more substantial reductions in carbon risk, covering both overall carbon emissions and their scopes, compared to those not using PCBs.

PCBs share the same objective as green bonds, aiming to protect the environment. However, the scope and application of PCBs differ from those of green bonds [[24], [25], [26]]. PCBs specifically focus on reducing pollution by including projects related to the quality of air, water, soil, etc. In contrast, green bonds involve a broader range of sustainability-related projects, such as renewable energy, energy efficiency, biodiversity preservation, and other green initiatives. PCBs are also often used for projects related to environmental infrastructure, waste management, sewage systems, etc [[27], [28]]. On the other hand, green bonds can be applied to various sectors, including energy, transport, sustainable agriculture, and other areas promoting environmental responsibility.In terms of Certification and Transparency, PCBs may be issued following specific standards and certifications related to pollution control, but they may not adhere to the same criteria as green bonds. Green bonds are often issued in accordance with international standards, such as the Green Bond Principles, establishing guidelines to ensure the transparency and environmental credibility of financed projects. We focused on the effect of PCBs in electric utilities rather than the impact of green bonds for several reasons. Electric utilities often prioritize reducing polluting emissions associated with electricity generation, making PCBs more aligned with these environmental challenges. PCBs can also be used to finance the modernization of power generation facilities, focusing on adopting cleaner technologies and reducing emissions of air pollutants. Electric utilities often generate specific industrial wastes that can be minimized through PCBs, helping them comply with current environmental regulations, leading to reputational benefits and avoiding financial penalties. Finally, PCBs can fund research and development projects aimed at developing cleaner, more efficient technologies, crucial for the long-term sustainability of this industry.

The rationale for choosing U.S. electric utility firms is rooted in the relevance of carbon risk. On one hand, the focus on U.S. firms is driven by the nation's standing as the third-largest country in the world, after Russia and Canada, acknowledging global warming, notably along its coasts. Additionally, the U.S. holds the position of the second-largest emitter of GHG globally, accounting for 18 % in 2022, following China's 28 %. Moreover, the U.S. ranks among the top five carbon exporters, totaling $325 million tons of CO2 in 2021, following China, Russia, Germany, and South Korea. The U.S. market for CO2 is prominent, experiencing a compound annual growth rate of 4.9 % in revenue in the upcoming years. Notably, unlike many developed countries, the U.S. refrained from ratifying the Kyoto Protocol, which mandated governments to achieve a 5 % reduction in GHG emissions. Finally, PCBs are one of the earliest green financial instruments in the U.S. that have been issued by municipal authorities to fund pollution control facilities. These bonds offer several financial advantages, including tax-exempt revenue bonds and lower interest rates (Robert et al., 1975).

On the other hand, the electric utility industry is a compelling focus due to its significant contribution to GHG emissions, with electricity production being the largest contributor in the USA. In 2022, CO2 emissions from the U.S. electric power sector amounted to 1539 million metric tons (MMmt), constituting approximately 31 % of the total U.S. energy-related CO2 emissions of about 4964 MMmt. Electric utility firms rank among the top scope 1 and scope 2 emission-intensive industries [1]. Challenges faced by these firms include increased costs due to climate policies, heightened demands on the electricity grid resulting from decarbonization efforts, and substantial institutional challenges associated with collaboration with the gas, transportation, and water industries. Furthermore, capturing carbon emissions poses significant challenges, given the inadequate development of operable carbon capture technologies within this industry. Additionally, there is a strong reliance on carbon-free electricity resources, including renewable sources such as hydropower, photovoltaics, onshore and offshore wind, as well as emerging technologies like utility-scale solar thermal generating stations, geothermal generation, fuel cells, ocean wave, and current generation. Finally, electric utility firms repay PCBs using revenue generated from the facility or through rate-payer fees over the project or facility's lifespan.

This study contributes to the existing literature in several significant ways. *First*, it stands as the pioneer in examining the effect of PCBs on carbon risk. *Second*, most prior research on carbon risk has concentrated solely on overall carbon emissions [2]. Other studies have focused solely on direct carbon emissions (scope 1) [3,19,23]. However, for an accurate representation of a firm's carbon risk, considering other scopes (scopes 2 and 3) is essential due to their additional exposure implications. Therefore, this study extends prior research by encompassing all three scopes of carbon emissions. Moreover, it goes beyond existing studies by deconstructing the carbon risk variable, providing comprehensive insights into carbon risk strategy and enabling an assessment of which scopes of carbon emissions are more or less affected by PCBs. *Third*, from a methodological perspective, the study uses quantile regression to analyze the distinct responses of different quantiles of the carbon risk distribution to green financing through PCBs. This choice is made because estimating only the conditional mean of the response variable can be inappropriate when the data do not meet the assumptions required for an Ordinary Least Squares (OLS) regression analysis. The quantile regression method offers a more comprehensive understanding of the PCBs-carbon risk relationship.

The remainder of this paper is structured as follows. Section 2 presents the institutional background. Section 3 develops the hypotheses. Section 4 presents the research design. Section 5 reports and discusses the empirical findings, and Section 6 concludes the paper."

## 2 Institutional background

The United States has prioritized reducing carbon risk, aiming for a 45–50 % reduction in total GHG emissions by 2030 compared to 2005 levels. Despite being an original signatory, the U.S. chose not to ratify the Kyoto Protocol, citing concerns about its potential economic impact, perceived enforcement inadequacies, the Byrd-Hagel resolution requirements, incomplete negotiation information, long-term considerations, and conflicting policy preferences within the U.S. Congress.

In April 2016, the U.S. signed the Paris Agreement and formally ratified it in September of the same year through an executive order. As part of its commitment, the U.S. pledged $3 billion to the Green Climate Fund. However, following the Paris Agreement's effective date on November 8, 2016, the U.S. administration contested the scientific consensus on climate change and human involvement. An executive order was issued to overturn the Clean Power Plan and other environmental regulations, reaffirming the U.S.'s opposition to efforts aimed at mitigating climate change.

During the G7 summit in May 2017, the U.S. declined to reaffirm its commitment to the Paris Agreement, making it the only dissenting member among the G7 nations. The U.S. officially withdrew from the agreement on November 4, 2020. This withdrawal increased carbon pricing for other countries while decreasing its own carbon pricing. However, on January 20, 2021, the U.S. reinstated its participation in the Paris Agreement, reaffirming its dedication to collaborating with other nations to address the urgent global challenge of climate change.

Under the Paris Agreement, the U.S. developed an ambitious and attainable Nationally Determined Contribution (NDC) to limit global temperature increases to 1.5 °C and achieve net-zero global emissions by 2050. The NDC was formulated through a comprehensive sector-by-sector assessment led by the National Climate Task Force, involving relevant government departments, agencies, and domestic stakeholders. This assessment used a detailed bottom-up model, considering factors such as capital stock turnover, technology costs, and external research. It accounted for emissions reductions from federal actions and subnational efforts, emphasizing investments in efficiency, clean energy, electrification, methane leak mitigation, and more. These actions are anticipated to generate employment, enhance public health, and promote equity and environmental justice.

The U.S. aims to achieve net-zero emissions by 2050 by investing in innovative GHG reduction solutions. The net-net accounting approach will be used to track progress, making corresponding adjustments for international mitigation outcomes. This NDC aligns with the emission peaking and reduction goals outlined in the Paris Agreement, seeking a balance between human-made emissions and GHG removals in the latter half of the century. The process involved consultations with various stakeholders, ensuring diverse perspectives were considered. The exploration of additional emission reduction opportunities beyond the NDC and national inventory continues, emphasizing the United States' commitment to climate action, as initiated by Executive Order 14008.

## 3 Theoretical framework

Theoretically, the environmental innovation theory may elucidate the impact of PCBs on carbon risk. The environmental innovation theory, as proposed by [29], is aimed at comprehending how to advance sustainable development. This involves establishing business models, industrial practices, and technological innovations that actively contribute to environmental sustainability. The focus is on reducing environmental impact, covering GHG emissions, air and water pollution, and the consumption of natural resources. Additionally, the theory aims to ease the transition to more sustainable energy sources by fostering innovation in renewable energy, energy efficiency, and clean technologies. The theory seeks to stimulate a shift towards sustainable practices in various domains, addressing environmental challenges comprehensively. This involves implementing economic models, industrial practices, and technological innovations that not only reduce environmental impact but also facilitate the energy transition. This dual approach include curbing GHG emissions, mitigating air and water pollution, and minimizing resource consumption. Furthermore, it actively encourages innovation in renewable energies, energy efficiency, and clean technologies, thereby supporting the transition to more sustainable energy sources. In essence, the environmental innovation theory provides a holistic framework for promoting sustainability by addressing both environmental impact and the imperative shift towards cleaner, more sustainable energy sources [30].

The fundamental principles of the environmental innovation theory shed light on the PCB-carbon risk relationship.

*First*, innovation acts as a catalyst for change, being indispensable in leading the shift towards more sustainable practices. PCBs'funds can be channeled into research and development (R&D), fueling innovative solutions to limit carbon emissions. This include initiatives to optimize resource utilization, develop cleaner technologies, and implement sustainable operational practices, thereby mitigating carbon risk.

*Second*, the theory asserts that environmental challenges demand adapted solutions beyond generic approaches, and one such remedy is green financing through PCBs.

*Third*, the pivotal role of R&D in generating environmental innovations is emphasized.PCB funds can be allocated for R&D initiatives, aiming at pioneering solutions to reduce carbon emissions. This includes initiatives to optimize resource utilization, advance cleaner technologies, and institute sustainable operating practices, all contributing to mitigating carbon risk.

*Fourth*, economic incentives have a substantial impact, pushing companies to innovate in the environmental domain. Mechanisms like tax benefits, subsidies, and commercial opportunities serve as incentives. In this context, PCBs present a range of economic incentives, encouraging companies to reinforce their commitment to sustainability and cut down on carbon risk.

*Fifth*, the theory acknowledges the pivotal role of stakeholders—consumers, investors, governments, and NGOs—in shaping companies' environmental innovation decisions. The adoption of PCBs enables companies to showcase their dedication to stakeholders, fostering a sustainability-friendly environment and managing risks tied to carbon emissions.

*Finally*, the environmental innovation theory emphasizes holistic approaches as imperative, necessitating a comprehensive view of the business value chain—from product design to end-of-life. PCBs can guide companies towards holistic practices right from the product design phase, integrating sustainable materials, minimizing the carbon footprint from the production stage, and designing products with recyclability or reusability in mind [31].

## 4 Hypotheses development

### 4.1 Carbon risk and PCBs

The impact of carbon risk is influenced by green financing facilitated through PCBs, as it leads to a reduction in polluting activities. Consequently, a decrease in the carbon footprint of firms is observed. Environmental strategies, which stakeholders such as regulators, governments, and organizations aim to exploit, may intentionally encourage the adoption of green finance [32]. This encouragement, in turn, results in firms being more inclined to invest in ecological projects, with PCBs offering financial advantages as a solution.

Carbon risk is mitigated by PCBs through three functions: the financing function, resource allocation function, and promotion of innovation.

*First*, the financing function of PCBs involves municipal bonds issued by local authorities or public companies. On one hand, these PCBs cover pollution prevention expenses, facilitating green investment. On the other hand, the California Pollution Control Financing Authority (CPCFA), the primary body issuing PCBs, actively participates in green financing. It offers California firms a viable green solution for environmental projects. Consequently, through the financing function of PCBs, CPCFA is recognized as California's inaugural "green bank."

*Second*, the reduction of carbon risk is facilitated by PCBs through the resource allocation effect. These bonds channel more financial resources into environmental projects, creating a more favorable financing environment with low-interest rates and tax-exempt revenue [33]. PCBs direct a significant portion of these resources towards polluting industries, particularly electric utility firms. Despite their high profitability, these firms carry substantial carbon risk. PCBs effectively manage the flow of financial resources. Additionally, unlike other green financing tools such as green credit, which restrict the credit availability for polluting firms [34], PCBs alleviate credit constraints. This is because these bonds are typically sold to institutions outside the company's traditional lenders. In exchange for these financial resources, firms are required to invest in pollution control equipment.

*Third*, the impact of PCBs on carbon risk is evident through their role in promoting innovation. Under the guidance of the CPCFA, PCBs facilitate the flow of additional financial resources to firms. Serving as a conduit bond issuer for private pollution control facilities with public benefits, CPCFA supports electric utility firms in enhancing their green innovation capabilities. Within this framework, the green bond structure plays a vital role at various stages of innovation. During the pre-issuance stage, an independent technical survey ensures that projects align with PCB's principles regarding environmental and clean energy policies. Post-issuance, there is a thorough examination of the use and management of PCB proceeds. Moreover, periodic assessments of projects are conducted to verify that they are meeting their innovation objectives. Simultaneously, PCBs alleviate financial constraints faced by many firms. For instance, in numerous cases, the costs associated with green bond verification or certification can be reimbursed.

PCBs contribute to the reduction of carbon risk, and we contend that such green financing achieves success in our context. We argue that PCBs offer significant benefits to electric utility firms for two reasons. *First*, to secure PCB financing, electric utility firms must actively commit to reducing carbon risk. This commitment results in reduced compliance costs and waste production. Furthermore, because electric utility firms are under intense public scrutiny, PCB financing minimizes or eliminates potential liabilities, fines, penalties, and clean-up costs [35]. Consequently, electric utility firms using green financing through PCBs are successful in obtaining financial support for environmental projects. Additionally, within the electric utility industry, this leads to the implementation or reinforcement of materials recovery facilities, landfills, waste-to-energy facilities, qualified solid waste or hazardous waste disposal projects, waste recovery facilities, the purchase of collection vehicles and residential waste containers, as well as water furnishing facilities and wastewater treatment facilities. All these initiatives contribute to the reduction of carbon risk. *Second*, the electric utility industry faces amplified stakeholder pressures and heightened scrutiny regarding their carbon risk [36,37]. These pressures tend to choose green financing over conventional methods because stakeholders are determined to observe low carbon risk and are more inclined to closely monitor its outcomes. Therefore, the following hypothesis is formulated.

Hypothesis 1 Pollution control bonds reduce carbon risk by decreasing overall carbon emissions in electric utility firms.

### 4.2 Individual carbon risk and PCBs

Each aspect of electric utility operations contributes differently to the various emissions scopes. Therefore, it would be appropriate to investigate the impact of PCBs on each carbon emissions scope individually, considering the distinct characteristics and sources associated with each. Every dimension addresses different aspects of GHG emissions related to a company's activities. Approaching this issue separately allows for a more in-depth understanding of the relationship between PCBs and carbon risk.

Scope 1 involves direct emissions from sources under the company's control, such as fuel combustion and industrial processes. In the case of electric utilities, Scope 1 emissions are linked to the combustion of fossil fuels for electricity generation. Actions to reduce these emissions may involve adopting cleaner technologies, improving energy efficiency, and transitioning to more sustainable energy sources.

Scope 2 deals with indirect emissions associated with the production of purchased energy, such as electricity and heat. In the context of electric utilities, indirect emissions (Scope 2) are often influenced by the energy mix used to procure electricity on the market. Strategies can focus on purchasing renewable energy, enhancing the energy efficiency of facilities, and decreasing electricity consumption [30].

Scope 3 covers a broad array of indirect emissions from the value chain, including suppliers, customer product use, upstream and downstream transport, and other sources. For electric utilities, indirect emissions (Scope 3) can be significantly influenced by the supply chain, especially the manufacture and maintenance of equipment. Strategies for this scope can concentrate on sustainable supply chain management, green product design, and adopting sustainable logistics practices.

The scopes exert different impacts on climate change. While direct Scope 1 emissions immediately impact climate change, indirect emissions from Scopes 2 and 3 also contribute but may exhibit distinct temporal, spatial, and sectoral characteristics. A separate analysis of the PCB-carbon risk relationship enhances the understanding of how actions in each scope contribute to climate change mitigation. Additionally, regulatory obligations and stakeholder expectations may vary for each scope. Separately studying the effect of PCBs on carbon risk ensures that companies meet specific standards for each emission type. Furthermore, stakeholders, including governments and customers, may have distinct expectations regarding emission reduction in each scope. For instance, governments, as regulators, may impose specific carbon emission standards and regulations for the electric utility industry, and these standards may vary according to each scope. Customers can also encourage electric utilities to influence their suppliers to adopt sustainable practices and reduce emissions throughout the supply chain (Scope 3).

Thus, within the context of electric utilities, studying the PCB-carbon risk relationship by focusing on each scope individually is interesting, as it takes into account the characteristics of each segment, the energy sources used, and associated emissions. This study tests which scope emissions are reduced through PCBs financing.

#### 4.2.1 Scope 1 and PCBs

Scope 1 carbon emissions, as defined by the GHG, cover direct GHG emissions from sources that the company controls or owns. These emissions may include those from fuel combustion at company facilities, fugitive GHG emissions, and other sources under the company's direct control.

PCBs can contribute to reducing Scope 1 carbon emissions in several ways. By using PCB financing, firms can be incentivized to invest in cleaner, renewable energy sources to enhance their operations. Electric utility firms could allocate these funds to develop wind farms, solar installations, hydroelectric power stations, or other renewable energy sources. This would gradually replace carbon-intensive energy sources, thereby reducing direct carbon emissions (Scope 1). PCB financing also empowers electric utility firms to convert existing facilities to lower carbon technologies. For instance, a coal-fired power station could be transformed to run on natural gas, consequently reducing direct carbon emissions. Additionally, with PCBs, electric utility firms could modernize their infrastructure, including power stations. Investing in cleaner, more efficient technologies can help lower carbon emissions per unit of electricity generated. Therefore, PCB funds can be directed towards projects aimed at improving the energy efficiency of electricity generation processes. This may involve adopting more energy-efficient technologies and implementing more efficient operating practices, thereby decreasing the amount of energy needed to produce a unit of electricity and, consequently, the associated carbon emissions.

Furthermore, some industries are encouraged to implement carbon capture and storage technologies. These technologies aim to extract CO2 emitted by industrial sources before its release into the atmosphere. PCB funds can be allocated to investments in clean carbon capture and storage technologies, capturing CO2emissions produced by electricity generation facilities before release into the atmosphere, thereby contributing to the reduction of direct emissions (Scope 1).

Although fugitive emissions may be less common in the context of electric utilities compared to certain industries such as oil and gas extraction, the chemical industry, or other gas-intensive sectors, there may be specific aspects related to emissions in this sector. PCBs encourage firms to make sustainable decisions such as the purchase and installation of low fugitive emission equipment. This includes the use of improved sealing technologies, specific construction materials, capture devices to minimize potential leaks, and R&D of alternative gases with low global warming potential.

PCBs also support the adoption of innovative technologies, such as advanced energy storage, smart grids, and other solutions that enable more sustainable electricity production and distribution. PCBs encourage electric utility firms to invest in transport electrification projects using renewable energy, which reduces carbon emissions from vehicle fleets, often included in Scope 1. Finally, PCBs often result from environmental regulations, especially in electric utility firms aiming to limit GHG emissions. By complying with these regulations, electric utility firms help reduce their direct Scope 1 carbon emissions.

In light of the preceding discussion, PCBs encourage electric utility firms to adopt more sustainable practices, improve their energy efficiency, and invest in technologies and processes that emit less carbon, thereby reducing the direct carbon emissions included in Scope 1. Thus, we formulate the first sub-hypothesis.

Hypothesis 1a Pollution control bonds reduce carbon risk by decreasing scope 1 of carbon emissions in electric utility firms.

#### 4.2.2 Scope 2 and PCBs

PCBs' funds can be directed towards investments in renewable energy sources, such as solar, wind, hydro, or other forms of clean energy, leading to a reduced reliance on carbon-intensive energy sources for electricity generation. This contribution helps to diminish indirect emissions (Scope 2).

PCBs incentivize electric utilities to procure green electricity from renewable energy producers, resulting in a greater proportion of the distributed electricity being generated from clean sources. This practice reduces the indirect emissions associated with the purchase of electricity (Scope 2). Electric utilities can use PCBs funds to establish partnerships with clean energy suppliers, encouraging the transition to cleaner energy sources adapted to the specific energy needs of companies. PCBs facilitate increased investment in modernizing electricity infrastructure and networks through the adoption of smart technologies, the implementation of more resilient electricity networks, and the reduction of energy losses during transmission and distribution, enhancing overall efficiency. Furthermore, PCBs support the development and adoption of advanced energy storage technologies, optimizing the management of electricity generated from intermittent sources like solar and wind power. This, in turn, reduces the need for backup power stations running on carbon-intensive fuels.

Lastly, electric utility firms can implement energy efficiency programs for their customers, a process facilitated by PCBs financing. These funds can be used to encourage consumers to reduce their electricity consumption, thereby helping in the reduction of indirect emissions.

In summary, PCBs play a significant role in reducing indirect carbon emissions (Scope 2) in electricity utilities. Through investments in clean energy sources, energy-efficient technologies, and energy efficiency initiatives, these companies can contribute to a transition towards more sustainable electricity production and distribution. Thus.

Hypothesis1b Pollution control bonds reduce carbon risk by decreasing scope 2 of carbon emissions in electric utility firms.

#### 4.2.3 Scope 3 and PCBs

PCBs play a vital role in reducing Scope 3 carbon emissions in electric utility firms in many ways. Electric utilities finance through PCBs to implement sustainable supply chain management practices by encouraging suppliers to adopt environmentally friendly practices and reduce their carbon intensity. PCB funds can be invested in the eco-design of energy infrastructure projects, such as designing and constructing electricity production facilities with a reduced carbon footprint and maximum energy efficiency. Additionally, PCBs reinforce educational programs aimed at raising customer awareness of more sustainable electricity use practices. Informing customers about the environmental impacts of their consumption can help reduce indirect emissions linked to the use of products and services (Scope 3). Through PCBs, electric utility firms are encouraged to adopt projects that promote the adoption of green electrification, such as the use of renewable electricity for applications and electric transport, thereby helping to reduce indirect emissions (Scope 3).

PCBs also reinforce waste management programs by extending the life of equipment, reducing the indirect emissions associated with the end-of-life of products. Furthermore, PCBs facilitate electric utility firms in optimizing efficient distribution networks, minimizing electricity losses during transmission, and adopting new sustainable technologies, such as smart grids, smart meters, and other solutions to make the entire electricity network more efficient and sustainable. Finally, electric utility firms can use the funds to reinforce relationships with stakeholders, including suppliers, customers, governments, and NGOs, by implementing joint initiatives to reduce indirect emissions.

According to the preceding discussion, PCBs play a crucial role in reducing indirect carbon emissions (Scope 3) in electric utilities by supporting projects and initiatives that target the entire value chain. By integrating sustainable practices into all levels of their operations, these companies can make a significant contribution to the transition to a more sustainable economy. We thus formulate the third sub-hypothesis.

Hypothesis1c Pollution control bonds reduce carbon risk by decreasing scope 3 of carbon emissions in electric utility firms.

## 5 Research design

### 5.1 Sample construction

In this paper, the primary objective was to investigate the influence of PCBs on carbon risk in electric utility firms. However, a potential issue related to endogeneity was identified. For instance, a company that was already committed to favorable environmental practices before using PCBs might be more inclined to use them. In this scenario, it might seem that issuing PCBs improves the company's environmental performance, when in reality, it was already improving before the green bonds were issued. To mitigate this potential bias, the Propensity Score Matching (PSM) methodology was adopted.

The methodology used in this study involved the creation of two distinct groups: the treatment group, consisting of electric utility firms utilizing PCBs, and the control group, composed of electric utility firms with similar characteristics that were not using PCBs. The sample was constructed by utilizing data on both PCB users' firms and non-PCB users available in the COMPSTAT database spanning from 2012 to 2022, including electric utilities during this period.

To tackle the challenges associated with matching and to identify an appropriate control group of non-PCB user firms, a treatment effect technique was employed. This method used all available information on covariates to estimate propensity scores, which were subsequently used as a unified continuous covariate for matching, following the approach outlined by Rosenbaum and Rubin [38]. The advantage of the PSM method lies in its ability to enable a comparison between companies that uses PCBs (the treatment group) and a control sample of similar cases that did not opt for PCBs. This approach facilitates attributing any observed effects to the issuance of green bonds itself.

To ensure comparability between the treatment and control firms, a reference sample was created to match the characteristics of the treatment group. Using a probit model, the propensity of companies to become PCB users was evaluated, considering various factors such as green innovation, leverage, financial performance, and firm size, all of which had been identified in previous studies [23].

Subsequently, a comparison was conducted between electric utility firms that used PCBs (the treatment group) and a set of matched control companies (the control group) within the same industry. Each company in the treatment group was paired with a counterpart in the control group exhibiting the closest propensity score. A maximum difference criterion of 3.0 % between propensity scores was imposed to ensure effective matching, following the methodology suggested by Lawrence et al. [39] and Khanchel and Lassoued [40]. This matching process resulted in 1331 pairs of observations, which, when merged with the full sample, yielded a PSM sample comprising 2662 observations. Consequently, the final dataset comprised 121 electric utility firms that had utilized PCBs, each matched with an equivalent number of control companies.

Finally, carbon risk data is measured by tons of CO2 equivalent (tCO2e) per year and is collected from the TRUCOST database.

### 5.2 Regression variables

#### 5.2.1 Carbon risk

The quantification of carbon risk relies on data from the TRUCOST database, serving as the primary data source. Two approaches to measuring corporate carbon risk (carbon_risk) have been outlined in previous studies [41,42].

*Absolute Carbon Emission Levels (scopes_lev):* This methodology is based on the absolute (unscaled) carbon emission levels expressed as the quantity of emitted carbon (tons of CO2-equivalent per year). Total CO2 emissions are utilized, categorized into three scopes:

Scope 1 of CO2 emissions (scope1_lev): Comprises direct emission sources owned or controlled by a company, such as energy generation within owned facilities (from any owned, non-renewable sources like generators), energy assets, and accidental or fugitive emissions like chemical and refrigerant leaks and spills.

Scope 2 of CO2 emissions (scope2_lev): Include indirect GHG emissions associated with the purchase of electricity, steam, heat, or cooling. This scope shows the effectiveness of a company in actively reducing its carbon risk, representing all the electricity or energy used to power equipment, office space, or various activities falling under scope 1 or scope 3 emissions.

Scope 3 of CO2 emissions (scope 3_lev): Includes all other emissions associated with a company's operations not directly owned or controlled by the company. This scope covers the energy used by utilities during transmission and distribution (T&D losses).

*Carbon Emissions Intensity (scopes_int)*: This methodology relies on carbon emissions intensity, defined as the ratio of carbon emissions to sales revenue. Carbon emissions intensity is expressed as tons of total CO2 emitted per million USD in total revenue (tCO2/$M). Similar to the first methodology, this metric is disaggregated into three categories:

Scope 1 of Carbon Emissions Intensity (scope1_int): represents the intensity of direct emissions.

Scope 2 of Carbon Emissions Intensity (scope2_int): indicates the intensity of indirect emissions associated with purchased energy.

Scope 3 of Carbon Emissions Intensity (scope 3_int): reflects the intensity of emissions associated with operations not directly controlled by the company.

Previous studies have indicated that higher emission levels correspond to greater carbon risk [43]. The distinction between total emissions and emissions intensity is crucial for two primary reasons. *First*, emissions intensity better signifies a firm's commitment to reducing emissions while maintaining overall productivity. *Second*, carbon risk must consider firms' emissions in proportion to their size, as cap-and-trade arrangements and carbon taxes are often linked to company size and revenue.

#### 5.2.2 TREAT

The dummy variable TREAT is introduced, taking the value of one if the firm uses PCBs in that year (Treatment group) and zero otherwise (Control group).

#### 5.2.3 Control variables

Several control variables linked to carbon risk in prior literature were included:

*Liquidity (LIQ):* Controlled through net cash flow from operations divided by the beginning period total assets. Sufficient cash flows are crucial for allocating resources to environmental projects, thereby reducing carbon risk [44].

*Research and Development (R&D) Intensity:* This variable influences carbon risk as it reflects the firm's innovative management strategies [45]. To control for this variable, total R&D expenses divided by total assets is used as a proxy.

*Asset Newness (CAPEX):* Highly correlated with environmental strategies, asset newness leads to cleaner and less polluting technologies, consequently reducing carbon risk ([46]; [15]. Asset newness is calculated as the ratio of capital expenditures to total sales revenue at the fiscal year-end.

*Financial Risk (RISK):* Financial risk accounts for higher monitoring in firms with high leverage [40], leading to more suitable environmental decisions and reduced carbon risk [15]. Financial risk is measured as total debt divided by total assets.

*Financial Performance (ROA):* Controlled by net income to total assets, financial performance ensures stakeholders' interests are better met by reducing carbon risk in high-performing firms [25].

*Firm Size (SIZE):* Accounting for larger companies exposed to higher political and regulatory pressures [47], firm size influences investments in green financing to mitigate carbon risk [43]. Firm size is determined using the natural logarithm of total assets.

A detailed presentation of variables is presented in Table A1 in APPENDIX A.

## 6 Empirical results

### 6.1 Descriptive statistics

Descriptive statistics for the variables are provided in Table 1. The average of the absolute carbon emissions levels is 4.666. Additionally, the mean of the three scopes of the carbon emissions levels ranges between 3.023 (scope2_lev) and 5.663 (scope3_lev), demonstrating significant variability around the mean values. Concerning the carbon emission intensity proxies, the mean of Scopes_int is 3.134, Scope1_int is 11.041, Scope2_int is 13.428, and Scope3_int is 10.994.

**Table 1:** Descriptive statistics.

Variable	Mean	Std. Dev.	Min	Max
Scopes_lev	4.666	1.411	1.477	8.429
Scope1_ lev	3.074	2.062	−1.585	8.361
Scope2_ lev	3.023	1.217	−0.582	5.942
Scope3_ lev	5.663	1.418	3.302	10.063
Scopes_int	3.134	2.31	−2.809	7.833
Scope1_ int	11.041	2.806	4.468	17.46
Scope2_ int	13.428	2.515	7.065	19.235
Scope3_ int	10.994	2.166	5.121	15.556
TREAT	0.5	–	0	1
LIQ	0.01	0.197	−1.035	0.316
R&D	0.171	0.212	0	0.795
CAPEX	0.043	0.05	0	0.255
RISK	0.58	0.272	0.098	1.695
ROA	−0.059	0.226	−1.051	0.27
SIZE	7.48	2.025	3.172	12.358

Table 2 presents the correlation matrix for all variables. The matrix illustrates that the correlation between independent variables is low, suggesting the absence of significant multicollinearity issues in this study.

**Table 2:** Correlation matrix.

Variables	(1)	(2)	(3)	(4)	(5)	(6)	(7)	(8)	(9)	(10)	(11)	(12)	(13)	(14)	(15)
(1) Scopes_lev	1.000														
(2) Scope1_lev	0.836^a^	1.000													
(3) Scope2_lev	0.800^a^	0.761^a^	1.000												
(4) Scope3_lev	0.868^a^	0.799^a^	0.793^a^	1.000											
(5) Scopes_int	0.597^a^	0.730^a^	0.446^a^	0.492^a^	1.000										
(6) Scope1_int	0.454^a^	0.735^a^	0.312^a^	0.358^a^	0.904^a^	1.000									
(7) Scope2_int	0.189^a^	0.307^a^	0.512^a^	0.198^a^	0.497^a^	0.392^a^	1.000								
(8) Scope3_int	0.483^a^	0.485^a^	0.341^a^	0.648^a^	0.651^a^	0.552^a^	0.319^a^	1.000							
(9) TREAT	−0.016^a^	−0.052^a^	−0.035^a^	−0.013	0.038	0.032	0.012	−0.031	1.000						
(10) LIQ	0.012	0.031	0.009	0.011	−0.045	−0.012	−0.046	−0.055	0.107^a^	1.000					
(11) R&D	−0.051^a^	−0.051	−0.093^a^	−0.104^a^	−0.057^a^	−0.020	−0.040	−0.091^a^	−0.144^a^	0.155^a^	1.000				
(12) CAPEX	−0.031	−0.006	−0.024	−0.044	−0.007	0.007	−0.020	−0.031	0.233^a^	0.117^a^	−0.246^a^	1.000			
(13) RISK	0.017	0.025	0.020	−0.034	0.043^a^	0.035	0.029	0.002	0.131^a^	−0.074^a^	−0.031	−0.002	1.000		
(14) ROA	0.039	0.050	0.015	0.005	−0.015	0.014	−0.048^a^	−0.046	0.124^a^	0.345^a^	0.143^a^	0.038^a^	−0.094^a^	1.000	
(15) SIZE	0.077^a^	0.115^a^	0.083^a^	0.012	0.073^a^	0.080^a^	0.054^a^	−0.027	0.360^a^	0.456^a^	0.044^a^	0.117^a^	0.141^a^	0.380^a^	1.000

a p < 0.05.

### 6.2 Inter-quantile regression results: PCBs and carbon risk

The following model is estimated based on the relationship between carbon risk and PCBs :

(1) $${\text{carbon}\_\text{risk}}_{i,t}{=\alpha_{0}+\alpha }_{1}{TREAT}_{it}+\sum \alpha_{i}{\text{CONTROLS}\;}_{i,t}+\sum YEAR+\varepsilon_{it}$$

This regression model is estimated separately for each carbon emission level variable (scopes_lev, scope1_lev, scope2_lev, scope3_lev) on one side, and each carbon emission intensity variable (scopes_int, scope1_int, scope2_int, scope3_int) on the other.

Equation (1) is estimated through quantile regression, chosen for its superior efficiency and robustness compared to OLS estimates. Originally proposed by Koenker and Bassett [48], quantile regression extends classical least-squares estimation. This method enables researchers not only to approximate the mean and median situated at the distribution center but also to construct a set of models for various functions. Each function characterizes the behavior of a specific point in the conditional distribution. Quantile regression is particularly valuable when the distribution lacks a standard form, such as in the case of an asymmetric distribution. Additionally, it serves as a consistent and robust estimation method, particularly when the error term exhibits heteroscedasticity and does not follow a normal distribution.

Quantile regression facilitates the examination of unique responses of carbon risk to PCBs across different quantiles of the distribution. This method enables us to determine the influence of green financing on various levels of carbon risk. The choice of quantiles is guided by insights from previous studies [46], commonly incorporating the median (τ = 0.5), the first and last deciles (τ = 0.05 and τ = 0.9), as well as the first and last quartiles (τ = 0.25 and τ = 0.75).

**GRAPH 1:**
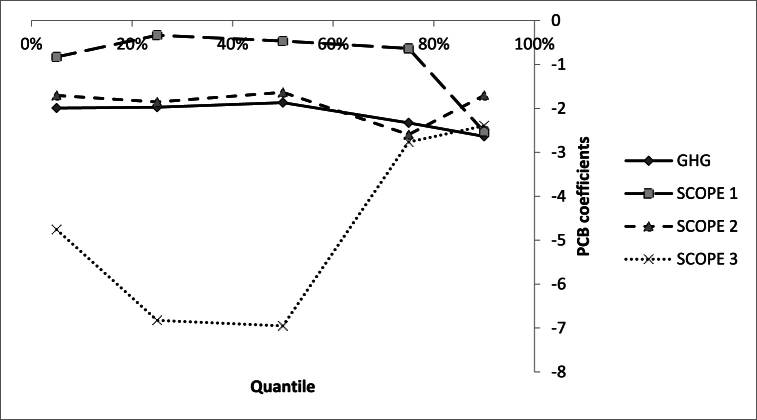
PCB-CO2 emission level.

Table 3, Table 4, Table 5, Table 6 and Graph 1 present the findings, including overall scopes, scope 1, scope 2, and scope 3 as the dependent variables (including both levels and intensities).

**Table 3:** Regression results with overall CO2 emissions level and intensity as the carbon risk measure.

	Panel A: Overall CO2 emissions level					Panel B: Overall CO2 emissions intensity				
	5	25	50	75	90	5	25	50	75	90
	(1)	(2)	(3)	(4)	(5)	(6)	(7)	(8)	(9)	(10)
TREAT	−1.992	−1.972^c^	−1.868^c^	−2.331^a^	−2.634^a^	−0.0739	−0.341^c^	−1.082^c^	−1.198^c^	−2.223^a^
	(1.037)	(0.383)	(0.635)	(1.221)	(1.385)	(0.582)	(0.196)	(0.553)	(0.679)	(0.681)
LIQ	−0.0917	−1.342	−0.237	−0.314	−0.205	−0.0894	−0.111	0.141	0.103	0.168
	(1.341)	(1.138)	(0.494)	(0.344)	(0.345)	(0.313)	(0.288)	(0.295)	(0.334)	(0.644)
R&D	−0.477	−1.338^a^	−1.663^a^	−1.125^a^	−0.985^c^	−0.743^a^	−0.666^a^	−0.302	−0.290	−0.974^b^
	(0.926)	(0.492)	(0.441)	(0.345)	(0.525)	(0.164)	(0.0896)	(0.214)	(0.254)	(0.494)
CAPEX	6.440^b^	0.200	1.091	−0.356	0.386	1.024	0.369	0.444	−0.281	0.655
	(3.208)	(2.619)	(1.185)	(0.917)	(1.120)	(0.746)	(0.503)	(0.707)	(1.121)	(1.600)
RISK	2.513^a^	1.206^b^	0.136	0.359^c^	0.105	0.233	0.120	0.277^b^	0.216	−0.0337
	(0.714)	(0.583)	(0.226)	(0.213)	(0.198)	(0.141)	(0.152)	(0.119)	(0.165)	(0.309)
ROA	0.363	−0.426	−0.545	0.247	0.0597	−0.0290	−0.352	−0.278	−0.490	−0.193
	(0.713)	(0.963)	(0.494)	(0.370)	(0.344)	(0.311)	(0.238)	(0.264)	(0.309)	(0.587)
SIZE	0.137	0.212	0.00884	−0.133	0.133	0.258^a^	0.149^a^	−0.0761	−0.315^a^	−0.0772
	(0.175)	(0.144)	(0.0924)	(0.0842)	(0.0845)	(0.0562)	(0.0387)	(0.0671)	(0.0725)	(0.124)
Year dummies	*yes*	*yes*	*yes*	*yes*	*yes*	*yes*	*yes*	*yes*	*yes*	*yes*
Observations	2584	2584	2584	2584	2584	2584	2584	2584	2584	2584

Robust standard errors in parentheses.

a p < 0.01.

b p < 0.05.

c p < 0.1.

**Table 4:** Regression results with Scope 1 CO2 emissions level and intensity as the carbon risk measure.

	Panel A: Scope 1 CO2 emissions level					Panel B: Scope 1 CO2 emissions intensity				
	5	25	50	75	90	5	25	50	75	90
	(1)	(2)	(3)	(4)	(5)	(6)	(7)	(8)	(9)	(10)
TREAT	−0.828^b^	−0.334^b^	−0.468^b^	−0.633^b^	−2.529^b^	−1.021^b^	−1.469^b^	−2.187^b^	−2.424^b^	−2.733^b^
	(0.412)	(0.163)	(0.238)	(0.255)	(1.200)	(0.495)	(0.791)	(1.099)	(1.201)	(1.344)
LIQ	−0.713	−0.669	0.0445	−0.221	−0.341	−0.570	0.216	0.159	−0.314	0.304
	(0.827)	(1.070)	(0.722)	(0.586)	(0.955)	(0.911)	(0.373)	(0.269)	(0.526)	(0.645)
R&D	−1.974^a^	−1.345^b^	−1.486^b^	−0.933^b^	−1.368	4.281^a^	2.711^a^	5.378^a^	4.496^a^	4.220^a^
	(0.568)	(0.553)	(0.687)	(0.404)	(1.029)	(0.502)	(0.473)	(2.047)	(1.024)	(0.627)
CAPEX	7.780^a^	2.092	0.927	−1.057	−0.326	1.575	0.518	−0.0932	−0.601	0.0340
	(1.972)	(2.029)	(1.284)	(1.651)	(2.426)	(2.012)	(0.948)	(0.509)	(0.717)	(0.836)
RISK	1.818^a^	1.104^a^	0.763^a^	0.686^a^	0.0953	0.237	−0.129	0.0606	0.267	−0.108
	(0.517)	(0.411)	(0.261)	(0.226)	(0.490)	(0.412)	(0.210)	(0.241)	(0.208)	(0.195)
ROA	0.251	−0.800	−0.752	−0.566	0.0647	−0.184	−0.319	−0.161	−0.259	−0.365
	(0.531)	(0.905)	(0.565)	(0.492)	(0.897)	(0.757)	(0.313)	(0.201)	(0.344)	(0.616)
SIZE	0.666^a^	0.145	0.0317	−0.253^c^	0.0277	0.635	−1.553^a^	−2.756^a^	−3.641^a^	−4.013^a^
	(0.164)	(0.171)	(0.134)	(0.132)	(0.143)	(1.228)	(0.480)	(0.294)	(1.030)	(0.632)
Year dummies	*yes*	*yes*	*yes*	*yes*	*yes*	*yes*	*yes*	*yes*	*yes*	*yes*
Observations	2584	2584	2584	2584	2584	2584	2584	2584	2584	2584

Robust standard errors in parentheses.

a p < 0.01.

b p < 0.05.

c p < 0.1.

**Table 5:** Regression results with Scope 2 CO2 emissions level and intensity as the carbon risk measure.

	Panel A: Scope 2 CO2 emissions level					Panel B: Scope 2 CO2 emissions intensity				
	5	25	50	75	90	5	25	50	75	90
	(1)	(2)	(3)	(4)	(5)	(6)	(7)	(8)	(9)	(10)
TREAT	−1.707	−1.852	−1.633^b^	−2.595^a^	−1.705^b^	−1.033	−0.384	−0.146^b^	−1.283^b^	−2.233^b^
	(1.069)	(2.169)	(1.095)	(0.407)	(0.828)	(2.110)	(0.381)	(0.572)	(0.612)	(1.200)
LIQ	−0.632	−1.177^b^	−0.451	−0.0494	0.136	0.524	−0.569	−0.412	−0.0566	0.172
	(1.416)	(0.532)	(0.454)	(0.442)	(0.596)	(1.159)	(0.387)	(0.304)	(0.329)	(0.472)
R&D	−1.711^a^	−1.337^a^	−1.894^a^	−1.288^a^	−1.680^b^	−0.536^c^	−0.120	−0.480^a^	−0.0988	−0.194
	(0.358)	(0.228)	(0.356)	(0.188)	(0.749)	(0.320)	(0.211)	(0.175)	(0.224)	(0.228)
CAPEX	1.124	0.190	0.361	0.464	2.244	1.567	0.838	0.148	−0.605	−0.726
	(2.581)	(0.659)	(1.526)	(1.165)	(2.110)	(2.614)	(0.590)	(0.831)	(0.655)	(1.586)
RISK	5.418^a^	4.661^a^	4.280^b^	2.705^a^	2.626^a^	2.511^a^	2.602^a^	2.683^a^	1.484^b^	1.614^c^
	(1.221)	(0.212)	(2.098)	(0.529)	(0.952)	(0.488)	(0.568)	(0.442)	(0.752)	(0.869)
ROA	0.358	−0.134	−0.245	−0.416	−0.128	−0.715	−0.363	−0.321	−0.351	−0.147
	(1.198)	(0.497)	(0.362)	(0.296)	(0.481)	(1.147)	(0.341)	(0.280)	(0.263)	(0.392)
SIZE	3.006^b^	2.169^a^	0.483	−1.080^b^	−1.504^c^	−0.959	−1.225^a^	−1.755^a^	−2.889^a^	−3.364^a^
	(1.303)	(0.523)	(2.083)	(0.473)	(0.850)	(1.140)	(0.217)	(0.449)	(0.822)	(0.931)
Year dummies	*yes*	*yes*	*yes*	*yes*	*yes*	*yes*	*yes*	*yes*	*yes*	*yes*
Observations	2584	2584	2584	2584	2584	2584	2584	2584	2584	2584

Robust standard errors in parentheses.

a p < 0.01.

b p < 0.05.

c p < 0.1.

**Table 6:** Regression results with Scope 3 CO2 emissions level and intensity as the carbon risk measure.

	Panel A: Scope 3 CO2 emissions level					Panel B: Scope 3 CO2 emissions intensity				
	5	25	50	75	90	5	25	50	75	90
	(1)	(2)	(3)	(4)	(5)	(6)	(7)	(8)	(9)	(10)
TREAT	−4.757^a^	−6.826^b^	−6.951^a^	−2.763	−2.394	−2.788^a^	−3.797^b^	−3.152^a^	3.196	1.891
	(0.501)	(2.945)	(2.245)	(1.940)	(1.741)	(0.430)	(1.826)	(0.279)	(1.510)	(2.035)
LIQ	−0.567	−0.324	−0.983	−0.207	0.663	−0.0160	0.180	0.269	0.191	0.596
	(0.974)	(1.394)	(0.864)	(0.831)	(0.764)	(0.282)	(0.558)	(0.332)	(0.749)	(0.533)
R&D	−1.127	−1.952^a^	−1.495^a^	−1.441^a^	−0.761	−0.0588	−0.685^b^	−0.977^a^	−0.170	−0.289
	(0.829)	(0.619)	(0.350)	(0.502)	(0.494)	(0.209)	(0.306)	(0.235)	(0.514)	(0.376)
CAPEX	−1.976	1.502	−0.344	−3.571	−3.322^a^	0.599	0.589	−0.227	−2.512	−2.733^b^
	(3.232)	(2.651)	(2.735)	(2.399)	(1.199)	(0.805)	(0.925)	(1.056)	(1.969)	(1.341)
RISK	1.693^b^	0.633	−0.500	−0.633	−0.478	0.240	0.0740	0.261	−0.378	−0.643^b^
	(0.689)	(0.888)	(0.448)	(0.475)	(0.323)	(0.210)	(0.245)	(0.168)	(0.460)	(0.320)
ROA	0.211	−0.865	−0.572	−0.830	−0.848	−0.175	−0.329	−0.441^c^	−0.546	−0.721^b^
	(0.778)	(1.105)	(0.703)	(0.756)	(0.728)	(0.209)	(0.386)	(0.250)	(0.631)	(0.307)
SIZE	1.178	4.239^a^	4.212^c^	2.017^a^	1.352^c^	4.953^a^	1.468^a^	1.540^a^	1.911^a^	3.433
	(2.790)	(0.928)	(2.237)	(0.553)	(0.740)	(0.664)	(0.406)	(0.272)	(0.402)	(2.277)
Year dummies	*yes*	*yes*	*yes*	*yes*	*yes*	*yes*	*yes*	*yes*	*yes*	*yes*
Observations	2584	2584	2584	2584	2584	2584	2584	2584	2584	2584

Robust standard errors in parentheses.

a p < 0.01.

b p < 0.05.

c p < 0.1.

Starting with Table 3 and examining the influence of PCBs on overall carbon emissions levels and intensity in Panel A and Panel B respectively, quantile regression shows a consistently positive relationship across all quantiles, confirming hypothesis H1.

Notably, the coefficients are more pronounced at the higher quantiles (specifically at the 75th and 90th quantiles for the level of carbon emissions and the 90th quantile for the intensity of carbon emissions). As a result, PCBs negatively impact both overall carbon emissions levels and intensity. Thus, electric utility firms financing through PCBs experience more substantial reductions in carbon risk compared to those not utilizing PCBs. The most significant effect of green financing is observed in electric utility firms with high carbon risk, highlighting the sensitivity of the company's overall carbon emissions levels to green financing.

Moreover, the coefficients show significantly lower values at lower quantiles and progressively increase at the higher quantiles. Consequently, it can be concluded that the impact of green financing through PCBs on carbon risk varies notably between the treatment group and the control group, especially in high-risk firms. One plausible explanation is that electric utility firms, using PCBs, enjoy numerous financial advantages, enabling their active participation in environmental projects aimed at mitigating carbon risk. However, firms with lower risk profiles typically exhibit a limited inclination to adopt product innovations, particularly those of a green nature, owing to direct public intervention and public ownership [49]. Additionally, stranded costs emerge as a notable characteristic of electric utilities, contributing to the lower proactivity of less risky firms in environmental projects. Lastly, the interests of utility investors and customers must be balanced by utility regulators, considering both regulatory and economic contexts, as well as the reasonableness of utility investment decisions.

The effect of PCB issuance on each category of carbon emissions levels (Scope1_lev, Scope2_lev, and Scope3_lev) and intensity (Scope1_int, Scope2_int, and Scope3_int) shows that the impact on scope 1 is the most substantial. Scope 1 includes direct carbon emissions originatingfrom sources owned or controlled by the firm, constituting most emissions footprints for most electric utility firms. As a result, electric utility firms focus their effort on diminishing this scope through projects funded by PCBs.

In Table 4, Panel A (for scope 1 of carbon emissions levels) and Panel B (for scope 1 of carbon emissions intensity), a consistently significant negative relationship is observed across all quantiles between PCB issuance and scope 1 of carbon emissions.These findings supported the hypothesis H1a. Similar to the overall carbon emissions, coefficients are notably higher at higher quantiles. Consequently, in comparison to firms that do not employ this green financing tool, the advantages of PCB issuance in reducing carbon risk are even more substantial when firms have high levels of carbon emissions. This highlights that green policies may result in increased costs for the electric utility industry, making them more likely to be implemented for high-footprint firms.

When considering scope 2 of carbon emissions levels and intensity as a measure of carbon risk (Table 5), similar results are observed. Although the coefficients in the 5th and 25th quantiles become insignificant, their signs remain negative. In other words, the provision of green financing continues to play a significant role in reducing carbon risk, specifically in the context of scope 2 of carbon emissions, at the middle to higher quantiles. Thus, the hypothesisH2b is supported.

In the case of scope 3 of carbon emissions levels and intensity (Table 6), the quantile regression shows a significant negative relationship between PCB issuance and carbon risk at the 5th, 25th, and 50th quantiles. Although the coefficients in the 75th and 90th quantiles are no longer statistically significant, their direction remains negative. These results indicate a clearly defined negative relationship between PCB issuance and scope 3 of carbon emissions levels and intensity, especially in the lower quantiles, confirming hypothesis H1c. In other words, compared to the control group, the issuance of PCBs benefits electric utility firms with low carbon risk (scope 3) rather than those with high carbon risk.

In summary, the findings show a robust and significant negative relationship between PCB issuance and carbon risk, notwithstanding a few insignificantly negative quantile coefficients. It can be concluded that compared to firms that do not issue PCBs, financing through PCBs effectively reduces carbon risk. The impact of PCB financing on carbon emissions can be explained in several ways. *First*, PCB issuance provides financial support for environmental innovation, procurement of green materials, and the implementation of new green technologies. *Second*, PCB issuance includes various projects financing part of pollution prevention expenses, with support from stakeholders, especially local authorities or public companies, enabling electric utility firms to invest in R&D for environment-related issues such as recycling infrastructures, composting, waste recovery, waste-to-energy, materials recovery, wastewater treatment, or water supply. *Third,* PCB issuance, through its financial advantages (tax exemptions, promotion of low-cost financing, etc.), helps in avoiding the high costs associated with traditional financing. Consequently, PCB issuance, as a green finance tool, promotes green policies by reducing environmental pollution and consequently lowering carbon risk [50].

The findings of our study align with previous research examining the effectiveness of green finance tools, particularly green bonds. For example, Gua et al. (2022) demonstrated that green finance tools generally contribute to reducing carbon emissions. Similarly, Xu et al. (2023) found that green finance significantly reduces carbon emissions. Studies focusing on green bonds also confirm their ability to reduce CO2 intensity compared to conventional bonds [23]. Flammer (2023) further indicated that the issuance of green bonds is associated with a decrease in emissions at the state level in the United States. Additionally, our results partially corroborate those of Lee et al. [51], suggesting that green bonds can reduce carbon emissions in China, although their effectiveness varies across regions.

### 6.3 Sensitivity analysis

During the sample period spanning from 2010 to 2022, two major international treaties for climate change, the Kyoto Protocol and the Paris Agreement, were adopted by nations worldwide. Following the ratification of these treaties, nations faced numerous challenges, one of which involved reducing GHG emissions. The impact of these treaties on the main findings was examined.

#### 6.3.1 The first phase of the Kyoto Protocol vs. the second phase of the Kyoto Protocol

Although the U.S. did not sign the Kyoto Protocol, the awareness of the need to reduce GHG emissions became crucial for many U.S. firms, leading to the adoption of proactive strategies [52]. To test whether our results held before and after the ratification of the Kyoto Protocol, the sample period was divided into two subperiods: the first phase of the protocol, from 2010 to 2012, and the second phase, from 2013 to 2020. Equation (1) was reestimated for each subperiod, and the results revealed some differences.

During the initial commitment period (the first phase), the effect of PCB issuance on carbon risk was marginally significant. This result can be attributed to the voluntary approach adopted by the United Nations Framework Convention on Climate Change, especially for many developing countries. Additionally, the U.S., the world's largest emitter of GHG, signed the Protocol but never ratified it, leading to reduced commitment from U.S. firms to GHG emissions reductions.

Estimations for the period from 2013 to 2020, corresponding to the second phase of the protocol, showed similar results to our main findings. This emphasized the heightened awareness of electric utility firms regarding GHG emission reductions. Notably, the goal of the first phase of the Kyoto Protocol, aiming for a 5 % reduction below 1990 levels, was not only achieved but surpassed by an average of 2.4 GtCO2e yr–1[53]. This success motivated more firms to exceed the target of the second phase, which aimed for an 18 % reduction.

Before commitment, commitment, Withdrawal, and Renewed Commitment to Paris Agreement.

The commitment of the U.S. to the Paris Agreement can be categorized into four stages: before commitment (2010–2015), commitment (2016–2019), withdrawal (2020), and renewed commitment (2021 and 2022). Our period was divided into these four subperiods based on this commitment timeline, and equation (1) was rerun for each subperiod. The results showed interesting insights.

*First*, during the 2010–2015 period, the effect of PCB issuance on carbon risk was found to be not significant for almost all measures of CO2 emissions. *Second*, in the 2015–2019 period, the effect was observed to be negative and highly significant, shedding light on the effectiveness of such commitment. *Third*, in 2020, the effect was observed to be positive; PCB issuance was seen to increase carbon risk. This was mainly explained by the strategy of many firms that focused on increasing their production after a reduction during the period of commitment to the Paris Agreement, rather than meeting the carbon risk reduction goals. *Fourth*, in the 2021–2022 period, the relationship was found to be almost non-significant. This aligns with some conclusions; the Paris Agreement lacks transparency, leading to some member states not presenting sufficiently ambitious GHG emission reduction targets.

### 6.4 Robustness check

To ensure the robustness of the results, alternative measures of the carbon risk variable were applied.

Following the recommendation of Qian and Schaltegger (2017)49 to use another corporate carbon performance as a proxy for low carbon risk, which includes emission reduction, the change in carbon emissions over two years was considered. The results remained consistent.

Furthermore, as suggested by Jung, Herbohn and Clarkson [19], gross property, plant, and equipment (PP&E) are utilized as the scalar instead of sales revenue. This change is motivated by the high correlation between sales revenue and economic performance, which can subsequently impact carbon risk. The results also remain consistent under this alternative measure.

## 7 Conclusion

This study investigated the relationship between PCBs issuance and carbon risk, using quantile regression methods on a sample of 242 U S. firms within the electric utility industry (121 firms utilizing PCBs and 121 matched non-PCB users) during the period 2012–2022.

The findings highlight that electric utility firms using PCB financing experience more substantial reductions in carbon risk, including overall carbon emissions levels and their scopes, compared to those abstaining from PCB utilization. Consequently, PCB issuance exhibits a robust and significant negative relationship with carbon emissions.

Exploring the differences between the treatment group (firms using PCBs) and the control group (firms not using PCBs) across various quantiles for each measure of carbon emissions showed significant distinctions, particularly for firms with middle to high footprints, as measured by overall CO2 emissions and scope 2 CO2 emissions. Moreover, the two groups exhibited notable differences across all quantiles of scope 1 CO2 emissions. Lastly, low-risk firms within the treatment group demonstrated a lower footprint, as measured by scope 3 of CO2 emissions, in comparison to their counterparts.

Additional tests were conducted to investigate the impact of global climate agreements on the relationship between PCBs issuance and carbon risk. *First*, the relationship was tested during the first and second phases of the Kyoto Protocol. The effect of PCBs issuance was marginally significant during the first phase of the protocol, whereas it became negative and highly significant during the second phase of this protocol. *Second*, the impact of the U.S. commitment to the Paris Agreement on the PCBs-carbon risk relationship was examined. The results indicated that no significant relationship was observed before commitment and renewed commitment. However, the negative effect was more acute during the commitment period, whereas during the withdrawal period, the relationship turned positive. Moreover, the results remained robust when alternative measures of carbon risk were used.

Taken together, our findings contribute significantly to the green finance literature and the emerging research stream on carbon risk.

*First*, we add impetus to the growing literature on carbon risk. Our results not only require researchers in the field of climate change to include green financing through PCBs as additional factors related to the reduction of carbon risk but also underline the importance of considering the carbon outcomes of any green practice and its impact on carbon emissions. This highlights the need to explore the outcomes and influence of other green practices in reducing carbon risk, challenging researchers to go beyond the traditional focus on firm performance, governance, and capital structure.

*Second*, we make a methodological contribution to the green finance and climate change literature by extending the traditional methodology. Our use of a quantile regression estimator shows its relevance, considering different impacts of PCBs issuance at various quantile levels of the firm's carbon risk. Consequently, our study provides a more comprehensive understanding of the impact of green financing on carbon risk.

*Third,* our results emphasize the crucial role of green instruments for financing in the context of strengthened carbon-related regulations and policies. Electric utility firms, sensitive to the consequences of climate change, appear influenced by carbon awareness, making green financing vital as they operate within a carbon-constrained environment.

*Fourth*, these findings have broader implications for regulators in shaping future policies regarding decarbonization in the electric utility industry. Anticipated trends, such as the addition of carbon-free electricity resources, can be highlighted, requiring regulatory support for environmental benefits beyond carbon emissions reduction, including reduced liquid and solid-waste emissions.

The study presents some limitations that could open new avenues for future research. *First*, although carbon emissions data are crucial for decision-making in green financing among electric utility firms, carbon risk disclosure is equally significant, potentially influencing carbon performance (49 ). Solely focusing on carbon risk provides an incomplete perspective, possibly disadvantaging firms with robust carbon management strategies and high disclosure levels. Our results suggest that considering carbon risk disclosure informs stakeholders and enhances their understanding of the future implications of carbon emissions. Future research could explore the impact of green financing on a firm's carbon risk strategy by incorporating a focus on carbon risk disclosure.

*Second*, the study focuses on green financing as a determinant of carbon risk. However, there may be a potential trade-off between debt and green financing for firms aiming to mitigate carbon risk, as lending institutions consider various carbon-related information in their lending decisions (Cogan et al., 2008). Future research could explore the joint effect of the two financing tools (PCBs and conventional) on carbon risk.

*Third*, this study focuses on scopes 1, 2, and 3 of carbon emissions. However, an organization's carbon risk can also include a fourth scope, Scope 4, which involves avoided emissions. These emissions relate to the quantities of GHG that are prevented due to the company's actions outside its perimeter. Scope 4 often accounts for emissions avoided through the financing of carbon offset projects, for example. Hence, future research investigating the impact of green financing issuance on Scope 4 of carbon emissions would be of interest.

## CRediT authorship contribution statement

**Imen Khanchel:** Visualization, Supervision, Project administration, Formal analysis, Conceptualization, Investigation. **Naima Lassoued:** Methodology, Investigation, Data curation. **Cyrine Khiari:** Writing – original draft, Validation.

## Declaration of competing interest

The authors declare that they have no known competing financial interests or personal relationships that could have appeared to influence the work reported in this paper.

## Figures and Tables

**Table A1:** Variable definitions

Variables	Notation	Description
**Dependent variables**		
Total carbon emission	scopes_lev	Absolute (unscaled) carbon emission levels
Scope 1 carbon emission	Scope1_lev	Absolute (unscaled) carbon direct emission sources owned or controlled by a company,
Scope 2 carbon emission	Scope2_lev	Absolute (unscaled) carbon indirect emission sources associated with the purchase of electricity, steam, heat, or cooling.
Scope 3 carbon emission	Scope3_lev	Absolute (unscaled) carbon indirect emissionsfromall other sources associated with a company's operations not directly owned or controlled by the company
Total carbon emission intensity	scopes_int	The ratio of carbon emissions to sales revenue
Scope 1 carbon emission intensity	Scope1_int	The ratio of carbon direct emissions to sales revenue
Scope 2 carbon emission intensity	Scope2_int	The ratio of carbon indirect emissions to sales revenue
Scope 3 carbon emission intensity	Scope3_int	The ratio of other emissions to sales revenue
**Independent variables**		
PCB financing	TREAT	A dummy variable that takes one if the firm use PCBs in that year (Treatment group) and zero otherwise (Control group).
**Control variables**		
Liquidity	**LIQ**	The ratio of net cash flow from operations to total assets.
Research and Development	**R&D**	The ratio of total R&D expenses to total assets
Financial Risk	**RISK**	The ratio of total debt to total assets.
Financial performance	**ROA**	The ratio of net income to total assets
Firm size	**SIZE**	The natural logarithm of total assets
